# Synergism of endophytic *Bacillus subtilis* and *Klebsiella aerogenes* modulates plant growth and bacoside biosynthesis in *Bacopa monnieri*

**DOI:** 10.3389/fpls.2022.896856

**Published:** 2022-08-04

**Authors:** Namita Shukla, Deepti Singh, Arpita Tripathi, Poonam Kumari, Rahul Kumar Gupta, Shiwangi Singh, Karuna Shanker, Akanksha Singh

**Affiliations:** ^1^Division of Crop Production and Protection, Central Institute of Medicinal and Aromatic Plants, Lucknow, India; ^2^Division of Phytochemistry, Central Institute of Medicinal and Aromatic Plants, Lucknow, India; ^3^Academy of Scientific and Innovative Research (AcSIR), Ghaziabad, India

**Keywords:** *Bacopa monnieri*, bacosides, endophytes, mevalonate pathway, phenylpropanoid pathway, lignin

## Abstract

*Bacopa monnieri* is the main source of pharmaceutically important bacosides; however, the low content of these molecules *in planta* remains a limiting factor for fulfilling the industrial requirement. The accumulation of secondary metabolites can be enhanced in plants upon inoculation with endophytes. In this study, we isolated and analyzed the culturable endophytes associated with different plant parts. By analyzing their impact on plant growth parameters (*in vitro* and *in vivo*) and Bacoside A content, we found few candidates which increased bacoside accumulation significantly. Finally, two promising endophytes namely *Bacillus subtilis* (OK070745) and *Klebsiella aerogenes* (OK070774) were co-cultivated with *B. monnieri* cuttings singly and in combination mode to clarify their effect on bacoside biosynthesis and their accumulation in *B. monnieri* shoot. Consortium-inoculated plants significantly enhanced the plant biomass and Bacoside A content with respect to single inoculation. The results of real-time quantitative (RT-PCR) revealed significant accumulation of bacoside biosynthetic pathway transcripts (*HMGCR, PMVK, FDPS, SQS*, and β*-AS)* in the case of plants inoculated with microbial combination, while the single inoculation of *B. subtilis* diverted the plant’s machinery toward the synthesis of phenylpropanoid genes like *CCR, CAD, CHS*, and *HST.* In addition, higher expression of *MYB 2* and *WRKY 1* transcription factors in combinational treatment points out their probable role in better physiological and developmental processes. Altogether, this is the first study on *B. monnieri*-endophyte interaction showing improvement in the accumulation of bacoside A by modulating various genes of metabolic pathway and thus suggests an effective “green approach” for augmenting *in planta* production of pharmaceutically important bacosides.

## Introduction

*Bacopa monnieri* (L.) Pennell (Family: Scrophulariaceae), an economically important medicinal plant, is used in Ayurvedic System of medicine since 5000 BC for improving memory, attenuating mental deficits, treating epilepsy, and reducing anxiety (Vishnupriya and Padma, 2017). The plant contains an extensive range of secondary metabolites ranging from alkaloids and herpestine to triterpenoid saponins. However, the major pharmacological actions of this plant are primarily due to the presence of bacosides which are therapeutically important dammarane class of triterpenoid saponins. Among the reported 12 analogs of bacosides; Bacoside A which is a mix of bacoside A_3_, bacopasaponin C, bacopaside II, and a jujubogenin isomer of bacopasaponin C is the major bioactive molecule reported to have preventive activity against Alzheimer’s diseases, chemically induced liver and cerebral toxicity, antidepressant, wound healing, etc. and is also the main reason for the pharmacological importance of this plant (Sukumaran et al., 2019).

The plant has two independent pathways for synthesizing the saponins of interest: mevalonate (MVA) pathway, which operates in cytosol, and the methyl-D-erythritol 4-phosphate (MEP) pathway, which operates in plastid. The product of both the pathways namely 3,3-dimethylallyl diphosphate and isopentenyl diphosphate acts as important mediators for producing the useful saponins in *B. monnieri* (Miziorko, 2011; Gupta et al., 2017a,b). The production of useful saponins through the abovementioned pathway is a result of action of several enzymes which bring about alterations in the structure of intermediates through oxidation, glycosylation, and substitution. In the MVA pathway, 3-hydroxy-3-methylglutaryl-CoA (HMG-CoA) intermediate formed is converted to MVA *via* 3-hydroxy-3-methylglutaryl-CoA reductase (*HMGCR*) using NADPH as the reducing agent. Later on in the same pathway, 5-phosphomevalonate kinase (*PMVK*) converts mevalonate 5-phosphate to mevalonate 5-diphosphate, the fifth step in the MVA pathway of isoprenoid biosynthesis. Further downstream the pathway, farnesyl diphosphate synthase gene (*FDPS*) initiates the catalysis of geranyl pyrophosphate and farnesyl pyrophosphate from isopentenyl pyrophosphate and dimethylallyl pyrophosphate. Ultimately, squalene synthase (*SQS*) and beta-amyrin synthase (β*-AS)* genes play a key role in synthesizing saponins of interest in the plants (Jeena et al., 2017).

Both plant yield and production of secondary metabolites are under the governance of multiple genes. For crop improvement, genetic manipulation, overexpression of key regulatory genes, and development of transgenic are a preferred choice. However, high production cost, acceptability by the society, and sustainability act as a roadblock for the implementation of such techniques (Kumar et al., 2020; Bate et al., 2021). Furthermore, to fulfill the industrial requirement of bacosides, the wild natural population of *B. monnieri* is being injudiciously used which has brought it in the category of threatened plant list. As an alternative to the above options, the application of microbes is being looked upon as a sustainable approach for enhancing crop and secondary metabolite yield in various medicinal and aromatic plants (MAPs) (Pandey et al., 2016, 2018; Gupta et al., 2019; Mastan et al., 2019; Ray et al., 2019). Most of the studies have focused on the application of plant growth-promoting microorganisms (PGPMs). Our group has also elucidated the role of microbes in modulating the secondary metabolite pathway in different MAPs (Singh et al., 2016, 2019; Gupta et al., 2019). Since last few years, the attention has shifted toward harnessing the benefits of endosymbionts known as endophytes. They are reported to invade the plant system through natural openings like stomata or root hairs which, later on, disperse systemically, thereby colonizing the intercellular or intracellular spaces (Wu et al., 2021). The endophytes help the plants by helping in nutrient acquisition, block the entry of pathogenic microorganisms, provide stress tolerance, and enhance the secondary metabolites of pharmaceutical importance (Omomowo and Babalola, 2019; Wu et al., 2021). In addition, endophytic microbes produce therapeutically important bioactive molecules similar to the host plant like Taxol, azadirachtin, camptothecin, and podophyllotoxin (Gupta et al., 2020; Slama et al., 2021). However, their large-scale production through fermentation has numerous hurdles ranging from instability in gene expression to losing the trait during repeated sub-culturing (Kusari et al., 2014). Therefore, use of endophytes to increase the production of secondary metabolites in plants is being considered as a better approach than independent *in vitro* cultures.

In case of MAPs, the secondary metabolite content holds an important place, and the role of endophytes in the modulation of secondary metabolite biosynthetic in the host plant has been previously established in medicinally important crops (Pandey et al., 2016; Mastan et al., 2019; Ray et al., 2019). Recently, *Echinacea purpurea* interaction with an endophyte was shown to enhance the secondary metabolites of therapeutic properties in this medicinal plant (Maggini et al., 2017). Likewise, tanshinone biosynthesis was enhanced in roots of *Salvia miltiorrhiza* when treated with an endophytic fungus *Chaetomium globosum* D38 and *Mucor circinelloides* DF20 (Zhai et al., 2018; Chen et al., 2021). Apart from single endophyte inoculation, co-inoculation studies involving different groups of microorganisms have also shown promising leads in benefiting the plants (Ray et al., 2019; Singh et al., 2021). In a study, a consortium of *Trichoderma viride* and *Glomus mosesae* showed significant enhancement in photosynthetic parameters and phosphorus content in *Rauvolfia serpentina* (L.) Benth (Kaushish et al., 2012). Recently, an amalgamation of four endophytes comprising of *Bacillus subtilis, Burkholderia* sp., *Bacillus licheniformis*, and *Acinetobacter pittii* significantly enhanced the yield of artemisinin than the individual microbes (Tripathi et al., 2020).

Considering the availability of scanty information available on endophytes-*B. monnieri* interaction efforts were made to identify, characterize, and study the impact of promising endophytic monocultures, as well as their synergistic combinations on plant productivity, *in planta* Bacoside A content. In addition, genes of bacoside and phenylpropanoid biosynthetic pathways were also targeted to validate the information obtained through biochemical studies.

## Materials and methods

### Sample collection and isolation of the endophytes

The plants of *Bacopa monnieri “*CIM-Jagriti” variety were collected from the experimental farm of CSIR-Central Institute of Medicinal and Aromatic Plants (CIMAP), Lucknow, India. The fresh plants were cleaned with water for removing any adhered epiphytes, soil debris, or dust particles on the plant surface. Different tissues of the plant parts (roots, stems, leaves, and buds) were dissected, and surface sterilization was done using 3% (v/v) sodium hypochlorite for 3 min followed by washing with 70% ethanol for 1 min. Finally, the plant material was washed 3 to 4 times with sterile distilled water, and the surface moisture was removed using sterilized tissue paper. For isolation of bacterial and fungal endophytes, each sterilized plant tissue was homogenized in a sterile mortar and pestle and serially diluted (up to 10^–6^) with 100 μl of the homogenate. Each dilution prepared was spread on nutrient agar (NA) and potato dextrose agar (PDA), respectively, for isolation of bacterial and fungal cultures. The NA plates were kept at 28 ± 1°C for 48–72 h, while the PDA plates were incubated at 28 ± 1°C for 5–10 days. Pure endophytic cultures were obtained by doing single colony isolation for bacteria on NA plates, while mycelia from fungal cultures were placed on PDA plates. After obtaining pure culture, bacterial isolates were classified based on their on-plate phenotypes which included colony color, shape, size, opaqueness, texture, surface, edge, and height. Likewise, fungal cultures were classified on the basis of color and texture of the fungal mat. In addition, sterilization check of surface-sterilized tissue was done by pipetting out 100 μl aliquot from the final wash buffer and inoculating on NA and PDA plates. Samples were rejected if any microbial growth was observed on check plates, and accordingly, new tissue samples were recollected. All the purified cultures were preserved at −80°C in 50% glycerol stock.

### Biochemical characterization of endophytes for plant growth-promoting traits

Each of the bacterial and fungal endophytic isolates was screened for their biochemical and plant growth-promoting activities by using standardized procedures and selective growth medium. The indole acetic acid production by the bacterial and fungal isolates was determined as per the protocol of Gordon and Weber (1951) using 1% L-tryptophan as a substrate. To estimate the phosphate solubilization ability of the endophytes, Pikovskaya’s agar medium was used (Pikovskaya, 1948). The inoculated plates were kept for 2–7 days at 28 ± 2°C. The appearance of clear zone around the bacterial and fungal colonies formed due to the solubilization of phosphate confirmed the phosphate solubilization ability of the culture/s.

Nitrate reduction ability of the endophytes was detected as per the protocol of Buxton (2011). Likewise, nitrogen fixation ability of the isolates was confirmed by the luxuriant growth on nitrogen-free medium Jensen’s medium (Jensen, 1965). Hydrogen cyanide and ammonia production ability was tested following the standard procedures described by Lorck (1948) and Cappuccino and Sherman (2005). Zinc solubilization was estimated as per the protocol of Vazquez et al. (2000). For rapid screening of cellulase producers, the isolates were cultured on the agar medium containing 0.5% (w/v) carboxymethyl cellulose (CMC) (Ruegger and Tauk-Tornisielo, 2004). The ACC deaminase activity of the bacterial and fungal isolates was screened as per the method of Dworkin and Foster (1958) using DF (Dworkin and Foster) minimal salts medium, while siderophore production was studied by the method described by Schwyn and Neilands (1987).

### Preparation of bacterial and fungal inocula for greenhouse experiment

Preparation for bacterial inoculum was done by culturing the bacterial isolates in 100 ml Nutrient broth (NB) at 28°C for 24 h under shaking condition at 120 rpm, while in case of fungal endophytes, the cultures were grown in PDA plates for 5–8 days and kept in BOD incubator at 28°C. The bacterial cultures were harvested by centrifugation at 7000 rpm carried out at 4°C for 10 min. The obtained culture pellet was washed with sterile distilled water and then dissolved in 0.85% saline. The concentration of the suspension was measured to A_600_ = 1.0 using spectrophotometer. Prior to the application of the inoculum to the plants, 1 × 10^8^ colony-forming unit (CFU) per ml was maintained for bacterial suspension. For fungal spore suspension preparation, the selected fungi were allowed to grow for 5–8 days till they were fully sporulating. The spores were scraped with the help of spore scraper and suspended in distilled water. Before inoculating in the plants, the concentration of spores was maintained to 1 × 10^8^ spores/ml. The greenhouse pot trial was carried out in the glasshouse. Earthen pots (4cm × 30cm × 25cm) were filled with 2:1 v/v mixture of autoclaved soil and vermicompost and watered with distilled water from time to time. The soil used in the study was sandy loamy with pH 7.2, 0.41 EC dS m^–1^, organic carbon 4.40 g kg^–1^, 120.6 kg ha^–1^ N, 11.2 kg ha^–1^ available P, and 96.8 kg ha^–1^ available K. Before filling the above potting mixture in pots, it was previously sterilized by autoclaving at 15 lbs for 20 min at 121°C.

For microbial inoculation, the surface-sterilized terminal cuttings of *B. monnieri* (L.) Pennell cv. “CIM-Jagriti” variety were collected from the National Gene Bank for Medicinal Aromatic Plants located at CSIR-CIMAP, Lucknow, India. The cuttings were dipped in individual endophyte bacterial/fungal spore suspension in the abovementioned concentrations and kept in the same state for 2 h before transferring them in pots. The cuttings dipped in only sterile 0.85% saline were taken as control. Pots were maintained at 28°C ± 2°C in a glasshouse with natural photoperiod and light intensity, and watering with distilled water was done as per the requirement. For ensuring the presence of sufficient number of endophytes in the soil, the inoculation with the previously mentioned CFU was carried out again at 20th day after the first inoculation. The experiment was set up in randomized block design (triplicates for each treatment) and to minimize experimental errors plant sampling for all analyses was done at the same stage and same position of the leaves.

### Estimation of photosynthetic pigments and plant growth parameters

Chlorophyll a, chlorophyll b, and total chlorophyll content of the fully expanded leaves was measured by crushing 500 mg leaf tissue in 10 ml of 80% acetone at 4°C and quantified as per the protocol of Arnon (1949). The absorbance of the supernatant was recorded at 480, 663, and 645 nm against the solvent (acetone) blank. The content of chlorophyll a and chlorophyll b was calculated by the given formulas:


Chlorophylla(μg/ml)[Chll]A=(12.7×A)663-(2.59×A)645

Chlorophyllb(μg/ml)[Chll]B=(22.9×A)645-(4.7×A)663

Totalchlorophyll(μg/ml)=20.2(A645)+8.02(A663)

The sampling was done 90 days after transplanting, and the plants were harvested for recording fresh weight (FW) followed by shade drying of the same material for 3–4 days before recording the dry weight (DW).

### Evaluation of the impact of endophytes on Bacoside A content

Hundred milligrams of shade-dried leaf samples was pulverized for preparing the methanolic extracts. The extraction was repeated three times, and the pooled extracts were vacuum-concentrated before determining the Bacoside A concentration using high-performance liquid chromatography (HPLC) system (Shimadzu Prominence-i LC 2030C 3D Plus) (Gupta et al., 1998). Bacoside A (1 mg/ml) was used as reference standard, and the identification of Bacoside A content of *B. monnieri* plants was determined by comparing the retention times with reference standard at 205 nm.

### Compatibility testing and development of microbial combination from two promising endophytes

The compatibility among two endophytic cultures (Y and A5) enhancing the bacoside content was determined *in vitro* by the cross-streak method. The bacterial inocula were prepared by culturing the bacterial isolates in 100 ml NB at 28°C for 24 h under shaking condition at 120 rpm. The final concentration of each bacterium was adjusted to 10^8^ CFU/ml. About 100 μl of a single cell suspension was spread on NA plates and was left for drying for 30 min at room temperature. Other bacterial culture was streaked on previously dried NA plate in such a way that both the cultures crossed each other at a single place on the plate. The plates were then incubated at 28°C for 24 to 48 h for checking the compatibility. No zone formation at the juncture was considered as positive compatibility. For consortium development, bacterial isolates were individually grown in NB at 28°C for 24 h under shaking conditions at 120 rpm. The bacterial cultures were harvested at 7000 rpm for 10 min at 4°C by centrifugation. The obtained pellet was washed with sterile distilled water and then dissolved in 0.85% saline. The concentration of the suspension was measured to A_600_ = 1.0 using a spectrophotometer. In case of consortium, an equal amount of each bacterial suspension was added and mixed well before inoculating in plants as described previously.

### Morphological and molecular characterization of the two potential endophytes

Morphological features of the two endophytes (Y and A5) were determined using light microscopy (Leica, Germany) and scanning electron microscopy (SEM). For SEM analysis, 1 ml of each bacterial endophyte grown in NB broth was fixed in 7% formaldehyde and kept overnight at 4°C. To the above samples, 9 ml of phosphate buffer saline (PBS; pH 7.0) was added in each vial followed by filtration through 0.2-μm Millipore filter and washing with PBS. Later on, dehydration of bacterial cells till critical point with 25, 50, 70, and 100% ethanol solutions was done three times for 10 min each. The bacterial endophytic cells were mounted on SEM stubs, sputter-coated with gold, and viewed on a FEI Quanta 250 SEM, Netherland (Nowell and Parules, 1980).

The bacterial genomic DNA of both the endophytic cultures was isolated by CTAB procedure as per the protocol of Sambrook (1989). The quantity and quality of extracted DNA were also checked spectrophotometrically using Nanodrop 1000 spectrophotometer (Thermo Fisher Scientific). Polymerase Chain Reaction (PCR) reaction mixture of 25 μl consisted of 1.25 U Taq polymerase, 0.2 mM deoxyribonucleotide triphosphate (dNTPs), 2.5 mM MgCl_2_, 10 pmol of each primer, 2.5 μl of 10 × reaction buffer, and 1 μg of template DNA and sterile Milli-Q water. 16S rRNA gene amplification was done by setting the thermocycling conditions as denaturation for 5 min at 94°C, followed by 30 cycles at 94°C for 45 sec, 57.4°C for 45 sec, 72°C for 2 min, and a final extension step at 72°C for 5 min. The PCR product was visualized on 1% (w/v) agarose gel amended with 10 μg ml^–1^ ethidium bromide which was also purified through Nucleo-pore PCR Clean-up Gel Extraction kit (Genetix Biotech Asia Pvt. Ltd., India) as per manufacturer’s instructions for carrying out Sanger’s sequencing. Finally, the identity of the two selected endophytes was done by doing Sanger sequencing using BigDye Terminator v3.1 cycle sequencing kit (Applied Biosystems, United States) on a Genetic Analyzer 3130 9 L (Applied Biosystems, United States). The 16S rRNA partial sequence data were deposited in the GenBank public sequence repository at the National Center for Biotechnology Information (NCBI) for getting the accession numbers. Phylogenetic tree construction was done using the bootstrap neighbor-joining tree approach using Clustal W alignment program.

### Effect of single and co-inoculation on the foliar nutrient uptake

Total nitrogen (N) content in the aerial part was quantified 60 days post-microbial treatment through Kjeldahl method by digesting the tissue with H_2_SO_4_ + H_2_O_2_. The content of potassium (K) and phosphorus (P) in the air-dried ground shoot samples was assessed after wet digestion with HNO_3_ + H_2_O_2_ following the protocol of Singh et al. (2009).

### Effect of single and co-inoculation on phytochemicals and antioxidant profile

Leaves from each treatment were randomly plucked 60 days post-microbial treatment and were immediately frozen using liquid nitrogen. Catalase activity in the leaves of *B. monnieri* was estimated as per the protocol of Aebi (1984). Superoxide dismutase (SOD) enzyme activity was calculated by observing the changes in absorbance at 560 nm (Nishikimi et al., 1972).

Total phenolic content (TPC) was estimated using the Folin–Ciocalteu method based on the reduction of phosphotungstate–phosphomolybdate complex by phenolics to a blue reaction product (Zheng and Shetty, 2000). Free radical-scavenging activity was estimated using the method of Brand-Williams et al. (1995). The scavenging potential of the extract was estimated according to the following equation:


Scavengingpotential(%)=[(absorbanceabsorbancecontrol-)sample/absorbance]control*100

### Effect of single and co-inoculation on the lignin deposition

Transverse sections of stem were cut, mounted in glycerol, and visualized under a fluorescent microscope EVOS FL Cell imaging system (Thermo Fisher). Lignin-deposited cells autofluoresced as blue-colored cells under excitation by UV range light (Singh et al., 2016).

### Quantitative real time polymerase chain reaction (qRT-PCR) analysis

Total RNA was isolated from the leaves of inoculated and control plants using TRIzol reagent (Invitrogen). RNAse free DNase1 (Takara) was used to remove DNA impurity, and the concentration of RNA was determined using Nanodrop 1000 spectrophotometer (Thermo Fisher Scientific) followed by synthesis of cDNA using RevertAid First-Strand cDNA Synthesis Kit (Puregene) as per manufacturer’s protocol. Transcripts of 14 genes involved in bacoside and phenylpropanoid biosynthetic pathways were quantified with gene-specific primers (Supplementary Table 1) picked from a transcriptomic study based on *B. monnieri* (Jeena et al., 2017). qRT-PCR was performed in triplicates of each biological sample. PCR mixture included 5 μl Power SYBR Premix Ex Mix (Takara), 0.2 μl ROX Reference Dye, 1 μl of 10 times diluted cDNA template, and 300 nM primers (forward and reverse) in a total reaction volume of 10 μl. qRT-PCR conditions were set as initial denaturation at 95°C for 10 min, 40 cycles of denaturation for 15 s at 95°C, and final extension step at 60°C for 1 min each. Fluorescent signals were recorded and analyzed on an Applied Biosystems Step One PlusTM Real-Time PCR System. The specificity of RT-qPCR was evaluated by using melting curve analysis (Applied Biosystems) of all amplicons. The actin gene was used as reference transcript, and the Ct value of control plants was used as calibrator. The analysis of mean expression of selected/target genes and relative quantification was done by 2^–ΔΔCt^method (Livak and Schmittgen, 2001).

### Statistical analysis

Statistical analysis of the data was done using ANOVA applicable to completely randomized block design (RBD using SPSS version 16). Significant differences among different treatments were carried out using Duncan’s multiple range tests (DMRTs) at a significance level of *P* ≤ 0.05. Correlations matrix and principal component analysis (PCA) were made using the R software (R v.4.0.2) with corrplot and factoextra packages.

## Results

### Isolation and biochemical characterization of endophytes for plant growth-promoting microorganisms traits

**FIGURE 1:**
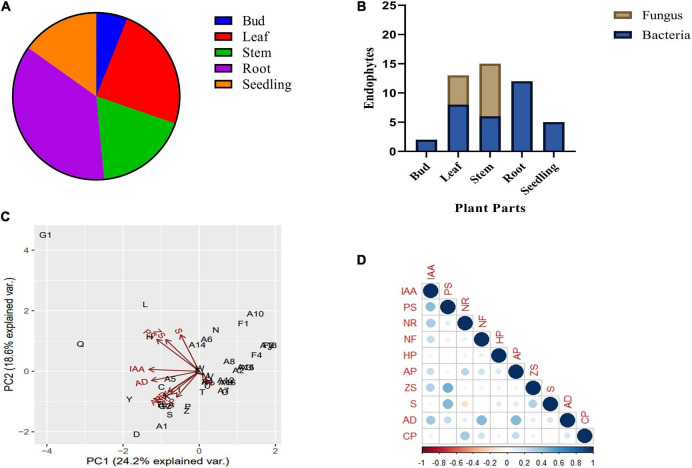
Endophytic bacterial and fungal diversity associated with different plant parts of *Bacopa monnieri* **(A)**; percentage distribution of bacterial and fungal endophytes associated with different plant parts **(B)**. Bi-plot ordination diagram of principal component analysis describing plant growth-promoting traits and functional activities of endophytes isolated from *B. monnieri*. PS, phosphate solubilization; NR, nitrate reduction; NF, nitrogen fixation; HP, hydrogen cyanide; AP, ammonia production; ZS, zinc solubilization; CP, cellulase production; AD, ACC deaminase activity; and S, siderophore production **(C)**. Pearson’s correlation matrix showing significant positive and negative correlations between different plant growth parameters. Blue circles represent positive correlation, red circles denote negative correlation, and the color intensity is proportional to the correlation coefficients **(D)**.

A total of 47 endophytes (34 bacteria and 13 fungi) were isolated from seedlings, buds, leaves, stems, and roots of *B. monnieri* plant (Supplementary Table 2). Out of the above, 10.64% endophytes were obtained from small seedlings, 4.25% from buds, 27.66% endophytes from leaves, 31.92% endophytes from stem, and 25.53% endophytes from the roots (Figure 1A). While talking about distribution pattern of endophytes, maximum number of endophytes were observed in stem followed by the leaf tissues (Figure 1B). All the bacterial and fungal endophytes were distinguished on the basis of different morphological characteristics of their colony (Supplementary Tables 3, 4). The isolated bacterial and fungal endophytes exhibited various biochemical and plant growth-promoting properties, and the compiled results of the study are presented in Table 1. Among all the endophytes, 17 isolates were found to be positive for IAA-producing trait while four isolates were found to solubilize phosphate in Pikovskaya’s agar with highest solubilization index being recorded for isolate G1 (Supplementary Table 5). Nitrate reduction trait was found in thirty-one isolates which was indicated by the formation of red violet color, while thirteen isolates were found to fix atmospheric nitrogen when grown on nitrogen-free Jensen’s medium (Table 1). In case of volatiles being produced by the endophytes, 15 isolates were found to be efficient HCN producers while forty-two were found to be positive ammonia producers (Table 1). Likewise, only one endophyte G1 was found to be zinc solubilizer with zinc solubilization index (ZSI) of 1.66 and solubilization efficiency (ZSE) of 266.67 (Supplementary Table 6). Siderophore production, ACC deaminase activity, and cellulase activity were found to be positive in ten, twenty-eight, and eleven isolates, respectively (Table 1). Furthermore, the ordination diagram using the above-screened plant growth-promoting traits revealed three major groups occupying two quadrants (Figure 1C). The first quadrant comprised of phosphate solubilizers, zinc solubilizers, indole acetic acid, and siderophore producers, while the second quadrant had ACC deaminase producers, nitrate reductase producers, nitrogen fixers, ammonia, and cellulase producers. Using correlation study, a positive correlation was observed between IAA production-phosphate solubilization traits and nitrate reductase producers-nitrogen fixers. In addition, a strong correlation was also found between phosphate solubilization with siderophore and zinc solubilization trait (Figure 1D).

**TABLE 1:** Plant growth-promoting properties of the endophytic isolates of *Bacopa monnieri.*

S. No.	Isolate		IAA	Phosphate solubilization	Nitrate Reduction	Nitrogen Fixation	HCN Production	Ammonia Production	Zn Solubilization	Sidero phore	ACC Deaminase	Cellulase Production
1	Control		−	−	−	−	−	−	−	−	−	−
2	A	Bacteria	+	−	+	+ +	−	+	−	−	+	+
3	B	Bacteria	+	−	+++	++	−	++	−	−	+	−
4	C	Bacteria	+ +	−	+ + +	−	+	++	−	−	+	−
5	D	Bacteria	+	−	+++	+++	−	++	−	−	+	+
6	E	Bacteria	+	−	−	−	−	+ +	−	−	+	−
7	F	Bacteria	+	−	+++	−	−	++	−	−	+	+
8	G1	Bacteria	+ +	+	+ +	−	−	+ +	+	+	+	−
9	G2	Bacteria	+	−	+++	−	−	++	−	−	+	+
10	H	Fungus	+	+	+ + +	−	−	+	−	−	+	−
11	I	Bacteria	+	−	−	++	−	++	−	−	+	−
12	J	Fungus	−	−	−	−	+	−	−	−	−	−
13	K	Fungus	+	−	+ + +	−	−	+ ++	−	−	+	−
14	L	Bacteria	+	+	−	−	++	++	−	+	+	−
15	M	Bacteria	+	−	−	+	+++	+	−	−	+	−
16	N	Bacteria	−	−	−	−	−	+	−	+	+	−
17	O	Bacteria	−	−	+ ++	−	+	+	−	−	−	+
18	P	Bacteria	+	−	++	−	+	+++	−	−	−	+
19	Q	Bacteria	+ +	+	+ + +	+	−	+ +	−	+	+	−
20	R	Bacteria	−	−	−	+	+	+ +	−	−	+	−
21	S	Bacteria	−	−	+++	+++	−	++	−	−	+	−
22	T	Bacteria	−	−	−	−	−	+ +	−	−	+	+
23	U	Bacteria	−	−	−	−	++	+++	−	−	+	−
24	V	Fungus	−	−	+ ++	−	−	+	−	−	+	−
25	W	Fungus	++	−	+++	−	−	+	−	−	−	−
26	X	Fungus	−	−	−	−	−	+ +	−	−	+	−
27	Y	Bacteria	+++	−	++	++	+	++	−	−	+	−
28	Z	Bacteria	−	−	+ ++	−	+ +	+ +	−	−	+	+
29	A1	Bacteria	−	−	+++	+	−	+++	−	−	+	+
30	A2	Bacteria	−	−	+	−	−	+ +	−	−	−	−
31	A3	Bacteria	−	−	−	−	+	++	−	−	−	−
32	A4	Bacteria	−	−	−	+	−	+ +	−	−	+	−
33	A5	Bacteria	−	−	−	++	−	++	−	+	+	+
34	A6	Bacteria	−	−	−	+	−	+	−	+	+	−
35	A7	Bacteria	−	−	+ ++	−	−	+	−	−	−	+
36	A8	Bacteria	−	−	−	−	−	+	−	−	+	−
37	A9	Bacteria	−	−	+ ++	−	+	+ +	−	−	−	−
38	A10	Fungus	−	−	−	−	−	−	−	+	−	−
39	A11	Bacteria	−	−	+ +	−	+	+	−	−	−	−
40	A12	Fungus	−	−	+ ++	−	−	+ +	−	−	−	−
41	A13	Fungus	−	−	−	−	−	−	−	−	−	−
42	A14	Bacteria	−	−	+ ++	−	−	+	−	+	+	−
43	A15	Fungus	−	−	−	−	+	++	−	−	−	−
44	A16	Bacteria	−	−	+ ++	−	+	+ +	−	−	−	−
45	F1	Fungus	−	−	−	−	−	+	−	+	−	−
46	F2	Fungus	−	−	−	−	−	−	−	−	−	−
47	F3	Fungus	−	−	−	−	−	−	−	−	−	−
48	F4	Fungus	−	−	−	−	−	+	−	−	−	−

+++ = Best performance; ++ = Average performance; + = Least performance, − = Absent.

### Impact of selected endophytes on the photosynthetic pigments

Out of the 47 endophytes isolated from the different parts of *B. monnieri*, seventeen isolates which showed promising *in vitro* plant growth-promoting traits were selected for carrying out the plant tests. Chlorophyll estimation was done in fresh green leaf samples, and the results varied significantly as highest increment in chlorophyll a was observed in plants treated with endophyte A16 followed by Y culture. Likewise, chlorophyll b was maximally enhanced in plants treated with endophyte Y followed by A7 culture (Table 2).

**TABLE 2:** Effect of selected endophytes on chlorophyll a and chlorophyll b content in *Bacopa monnieri* plants with respect to control set plants. Results represent mean ± standard error of three replicates.

S.No.	Treatments	Chlorophyll a (μg/ml)	Chlorophyll b (μg/ml)	Total chlorophyll (μg/ml)
1	Control	1.08 ± 0.13	2.12 ± 0.10	3.59 ± 0.77
2	C	3.26 ± 0.39	5.18 ± 0.39	9.48 ± 0.81
3	D	2.93 ± 0.27	4.14 ± 0.15	7.94 ± 0.90
4	F	2.08 ± 0.13	4.07 ± 0.28	6.89 ± 0.66
5	H	4.74 ± 0.98	4.43 ± 0.17	10.31 ± 0.86
6	G1	4.05 ± 0.28	7.01 ± 0.29	12.42 ± 0.82
7	L	2.93 ± 0.94	4.14 ± 0.37	7.94 ± 0.88
8	M	4.67 ± 0.79	6.2 ± 0.12	12.21 ± 1.13
9	N	3.31 ± 0.23	5.74 ± 0.57	10.16 ± 0.54
10	Q	4.18 ± 0.29	4.64 ± 0.84	9.91 ± 0.77
11	S	3.74 ± 0.72	5.78 ± 0.40	10.68 ± 1.28
12	W	1.21 ± 0.12	3.04 ± 0.38	4.76 ± 0.69
13	Y	5.20 ± 0.27	7.36 ± 1.01	14.11 ± 0.81
14	A5	2.63 ± 0.13	2.89 ± 0.13	6.21 ± 0.49
15	A6	3.47 ± 0.44	5.29 ± 0.13	9.83 ± 0.60
16	A7	4.39 ± 0.14	6.88 ± 0.73	12.66 ± 0.33
17	A14	2.22 ± 0.12	2.66 ± 0.22	5.48 ± 0.99
18	A16	5.29 ± 0.98	6.64 ± 0.34	12.54 ± 0.93

### Impact of selected endophytes on biomass, number of flowers, and Bacoside A content

**FIGURE 2:**
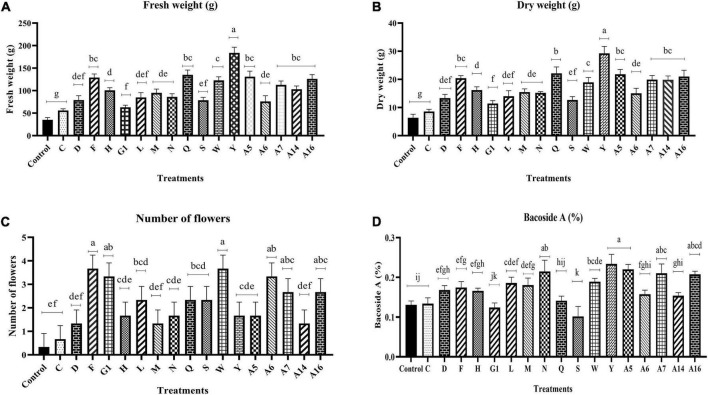
Effect of endophyte inoculation on fresh weight **(A)**, dry weight **(B)**, number of flowers **(C)**, and Bacoside A content **(D)** in 90 days old *Bacopa monnieri* plants. Non-inoculated plants were used as control. The results are expressed as means of three independent biological replicates. Different Duncan’s letters denote significant difference at *P* < 0.05.

The impact of endophyte inoculation on the *B. monnieri* plants was clearly visible in terms of enhancement of plant fresh biomass as significant increase ranging from 59.27 to 422.66% was observed in endophyte inoculated set in comparison with the control plants (Figure 2A and Supplementary Figure 1). The results followed a similar trend in case of dry weight as maximum significant increment was observed in plants inoculated with culture Y with least increase in plants treated with endophytic culture C (Figure 2B). The application of endophytes also had a significant impact on flowering status of the plants as endophytic cultures F, G1, W, A6, A7, and A16 significantly increased the number of flowers with respect to the control plants (Figure 2C). Likewise, promising effect of the endophytes was also observed on the Bacoside A content as maximum significant increase by 78.70% was observed in culture Y followed by A5 (Figure 2D). In addition to the two mentioned cultures, N, A7, and A16 too increased the bacoside significantly. However, looking into the cumulative effect of endophytes on plant growth parameters and Bacoside A content, A5 and Y cultures were taken up for further studies. In addition, to our surprise, one endophytic culture had a negative effect on Bacoside A content as it reduced its content with respect to the control set plants.

### Compatibility testing, and morphological and molecular characterization of two potential isolates

Based on the above results, cultures A5 and Y were found to be potential candidates for the development of synergistic combination. The cross-streak method results revealed that both the endophytes grew well together and did not hamper each other’s growth.

**FIGURE 3:**
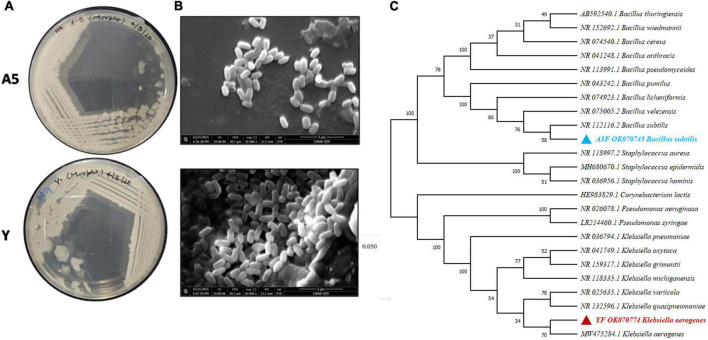
Morphological **(A)** and scanning electron micrographs showing the size and shape of bacterial endophytes, *Bacillus subtilis* (A5) and *K. aerogenes* (Y). The image of clusters of bacterial colonies was focused at 5-μm scale **(B)**. The 16S rRNA gene sequences of bacteria A5 and Y, as well as related bacteria in the genera *Bacillus* and *Klebsiella*, were used to create a maximum-likelihood phylogenetic tree with bootstrap values expressed as a percentage of 1000 replicates. *Corynebacterium lactis* strain HE983829.1 was used as an outgroup for making the phylogenetic tree **(C)**.

The morphological appearance recorded through light microscopy and SEM analysis revealed rod-shaped structure of both the bacterial endophytes (Figures 3A,B). The 16S rRNA gene sequence NCBI-BLAST result confirmed that bacterial endophyte A5 showed maximum similarity with *Bacillus subtilis* (NCBI accession number OK070745). Likewise, the 16sRNA gene sequence of endophytic culture Y showed maximum homology with *Klebsiella aerogenes* (NCBI Accession Number OK070774). Phylogenetic relationship of A5 and Y was analyzed using related sequences obtained from NCBI (National Centre for Biotechnology Information) GenBank, and the relationship has been depicted in Figure 3C.

### Effect of selected endophytes singly and in combination on foliar nutrient uptake, plant biomass, and Bacoside A content

**FIGURE 4:**
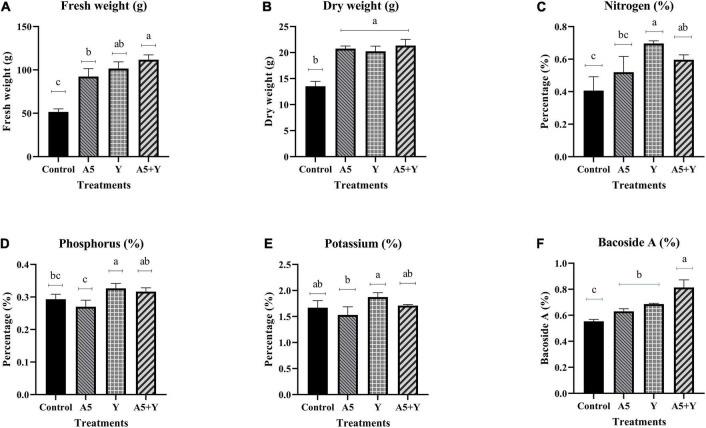
Influence of endophyte inoculation on fresh weight **(A)**, dry weight **(B)**, nitrogen content **(C)**, phosphorus content **(D)**, potassium content **(E)**, and Bacoside A **(F)** in 60 days old *Bacopa monnieri* plants inoculated with endophytes A5 and Y individually and in the form of consortium (A5 + Y). Non-inoculated plants were used as control. The results are expressed as means of three independent biological replicates. Different Duncan’s letters denote significant difference at *P* < 0.05.

The application of endophytic consortium significantly enhanced the fresh weight, dry weight, N, and Bacoside A content in *B. monnieri* plants. Inoculation with a consortium of *B. subtilis* and *K. aerogenes* increased the fresh weight and dry weight of *B. monnierri* plant by 117.08% and 57.63%, respectively, when compared to the control plants (Figures 4A,B). Significant increase by 46.63% was observed in case of N content in microbial combination treatment; however, insignificant effect of combination of endophytes was observed on K content (Figures 4C–E). Likewise, significant increment in Bacoside A content by 46.99% was observed in microbial consortium treated plants than that of control set plants (Figure 4F).

### Effect of selected endophytes singly and in combination on antioxidant status and lignification pattern

**FIGURE 5:**
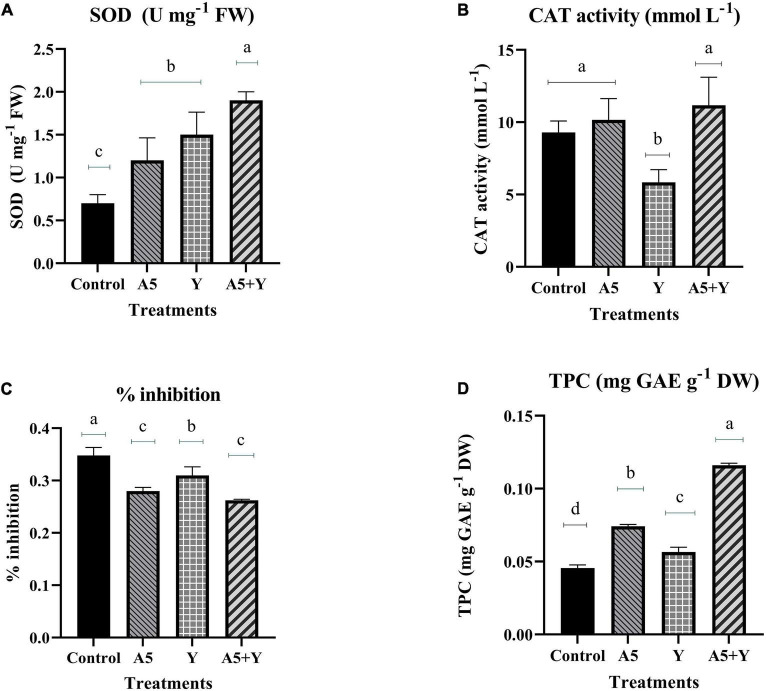
Influence of endophyte inoculation on superoxide dismutase enzyme **(A)**, catalase enzyme activity **(B)**, free radical-scavenging activity **(C)**, and total phenolic content **(D)** in 60 days old *Bacopa monnieri* plants inoculated with endophytes A5 and Y individually and in the form of consortium (A5 + Y). Non-inoculated plants were used as control. The results are expressed as means of three independent biological replicates. Different Duncan’s letters denote significant difference at *P* < 0.05.

To study the impact of selected individual endophytes and their combination on antioxidant status of *B. monnieri* plants, the activity of SOD and CAT enzyme was assessed (Figure 5). To our observation, there was a significant increase in SOD enzyme activity in all the microbial treatments; however, maximum increment by 171.42% was observed in endophytic consortium treatment (Figure 5A). In case of catalase (CAT) enzyme, insignificant effect was observed in all the microbial treatments with respect to the control plants (Figure 5B). The free radical-scavenging activity and TPC content in *B. monnieri* plants were significantly influenced by both monocultures and microbial combination. The highest significant% inhibition in free radicals was observed in microbial consortium treatment followed by A5 culture (Figure 5C). Likewise, TPC was also significantly enhanced in microbial consortium treatment (92.20%) with respect to control set plants. Among the single individual inoculations, *B. subtilis* performance was better than *K. aerogenes* and control plants (Figure 5D).

**FIGURE 6:**
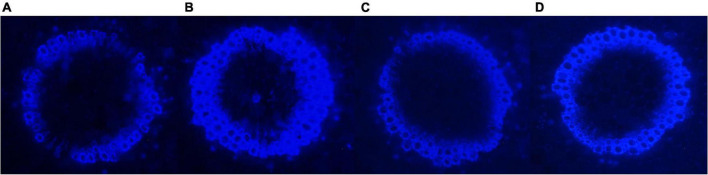
Influence of endophyte inoculation on lignification pattern in the stem of control **(A)**; *Bacillus subtilis* (A5) inoculated plants **(B)**; *Klebsiella aerogenes* (Y) inoculated plants **(C)**; and consortium (A5 + Y) inoculated **(D)** *B. monnieri* plants.

To envisage the effect of selected endophytes individually and their combination on the lignin deposition, stem sections of *B. monnieri* plants were visualized under fluorescent microscope (Figure 6). The maximum and uniform lignification pattern visible as a blue ring was observed in A5 followed by combination and Y treatment alone. However, stem sections of control plants had thinner and interrupted lignified cells which pointed out toward the role of microbes in strengthening the defense barrier of the plant system (Figure 6A).

### Quantitative real-time-PCR analysis of transcription factors, bacoside, and phenylpropanoid pathway genes

**FIGURE 7:**
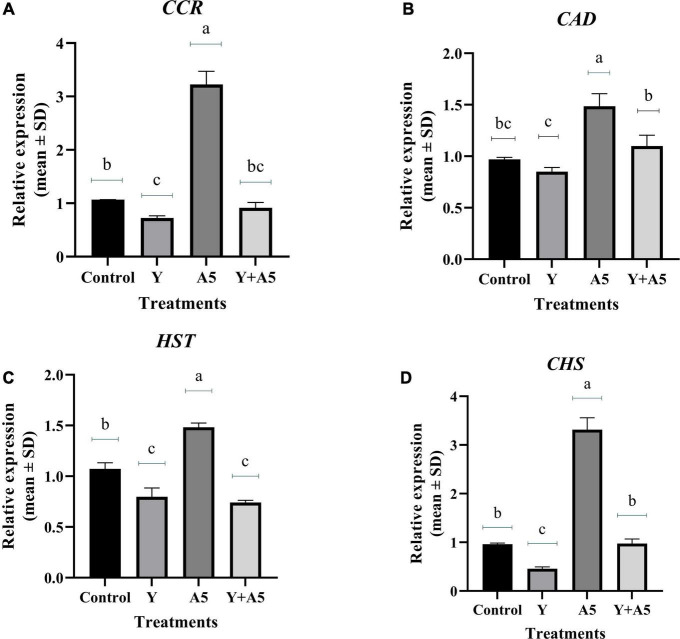
Influence of endophyte inoculation on the expression of genes involved in phenylpropanoid metabolism. Total RNA was isolated from the leaves of 60 days old *B. monnieri* plants inoculated with endophytes A5 and Y individually and in the form of consortium (A5 + Y), reverse-transcribed and used for expression analysis by SYBR Green-based RT-qPCR leaves on untreated plants were considered as control. Expression of *CCR* **(A)**, *CAD* **(B)**, *HST* **(C)**, and *CHS* **(D)** was analyzed. Actin was used as a reference transcript. Data are means ± SE (*n* = 3 biological replicates), and Y-axis represents relative expression which was calculated using 2^–Δ^ ^Δ^ *^Ct^* method. Duncan’s letter denotes significant difference at *P* < 0.05.

**FIGURE 8:**
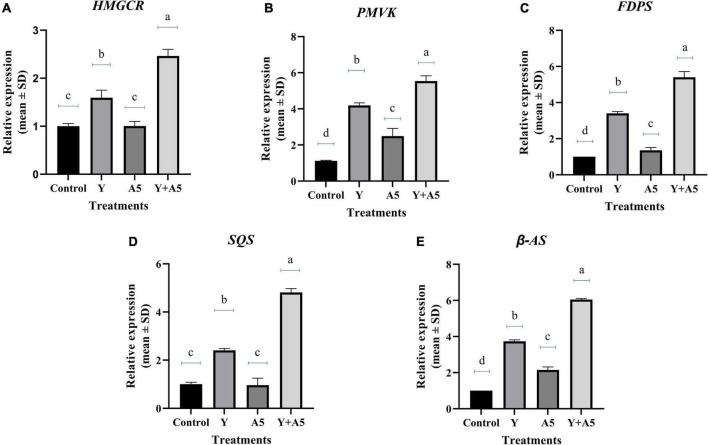
Influence of endophyte inoculation on the expression of genes involved in bacoside biosynthesis. Total RNA was isolated from the leaves of 60 days old *B. monnieri* plants inoculated with endophytes A5 and Y individually and in the form of consortium (A5 + Y), reverse-transcribed and used for expression analysis by SYBR Green-based RT-qPCR leaves on untreated plants were considered as control. Expression of *HMGCR* **(A)**, *PMVK* **(B)**, *FDPS* **(C)**, *SQS* **(D)**, and β*- AS* **(E)** was analyzed. Actin was used as a reference transcript. Data are means ± SE (*n* = 3 biological replicates), and Y-axis represents relative expression which was calculated using 2^–Δ^ ^Δ^ *^Ct^* method. Different Duncan’s letters denote significant difference at *P* < 0.05.

**FIGURE 9:**
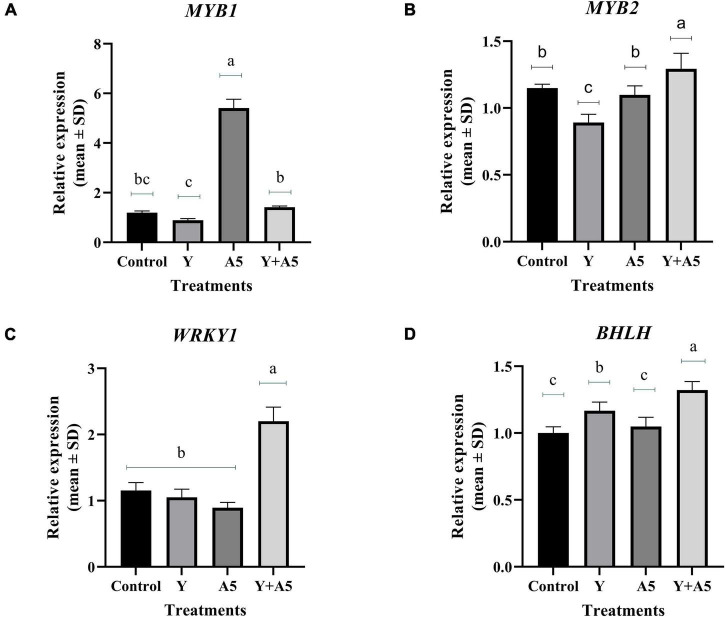
Influence of endophyte inoculation on the expression of genes involved in bacoside biosynthesis. Total RNA was isolated from the leaves of 60 days old *Bacopa monnieri* plants inoculated with endophytes A5 and Y individually and in the form of consortium (A5 + Y), reverse-transcribed and used for expression analysis by SYBR Green-based RT-qPCR leaves on untreated plants were considered as control. Expression of *MYB 1* **(A)**, *MYB 2* **(B)**, *WRKY* **(C)**, and *BHLH* **(D)** was analyzed. Actin was used as a reference transcript. Data are means ± SE (*n* = 3 biological replicates), and Y-axis represents relative expression which was calculated using 2^–Δ^ ^Δ^ *^Ct^* method. Different Duncan’s letters denote significant difference at *P* < 0.05.

To understand the mode of action of endophytes, the expression of several key genes of biosynthetic and phenylpropanoid pathways along with transcription factors was studied (Figures 7–9). A very interesting observation was recorded in the study as significant increase in phenylpropanoid genes was observed in the case of plants inoculated with *B. subtilis*, while an overall increase in MEV and MVA pathway genes was observed in microbial combination set. Significant increase in cinnamoyl-CoA reductase (*CCR)*, cinnamyl-alcohol dehydrogenase *(CAD)*, chalcone synthase (*CHS)*, and shikimate O-hydroxycinnamoyltransferase (*HCT)* genes was observed in *B. subtilis* [A5] inoculated plants in comparison with the control set plants (Figure 7). Likewise, a significant fold change in gene expression was recorded for *HMGR, PMVK, FDPS, SQS*, and β*-AS* genes in the case of plants inoculated with microbial combination. β*-AS* was one gene which showed enhanced expression in all the microbial treatments with respect to the control set plants (Figure 8). In case of transcriptional factors, *MYB* 1 was significantly enhanced in *B. subtilis* inoculated plants, while *MYB-2*, *WRKY-1*, and *BHLH* were most profoundly upregulated in treatment having both the microbes (Figure 9).

## Discussion

*Bacopa monnieri* plant houses diverse category of microbes as the culturable bacterial and fungal diversity varied significantly with respect to different plant parts. A notable observation pointed out toward more abundance of fungal endophytes in leaf and stem tissues which could be possible because of the presence of higher content of bacosides in the aerial tissues. Our speculation is in line with a study carried out on *Lycoris radiata* where amaryllidaceae alkaloids were hypothesized to play a crucial role in fine-tuning the balance and distribution of fungal endophytes in different plant parts (Zhou et al., 2020). Since the endophytes like other beneficial microbes enhance the plant growth by producing various plant growth-promoting traits, selection of potential strains for further studies was based on their ability for producing such traits. All the isolates except three cultures were found to be positive for one or the other plant growth-promoting traits in *in vitro* assay. Therefore, out of the 47 endophytic cultures, screening of cultures for *in vivo* plant growth promotion was based on the selection of functionally diverse group of microbes showing different plant growth-promoting traits as obtained in *in vitro* assays. The selection done was in accordance with the previous studies where endophytes with plant growth-promoting traits not only enhanced the plant productivity but also the secondary metabolites of pharmaceutical importance (Pandey et al., 2016; Ray et al., 2019; Mastan et al., 2020; Tripathi et al., 2020). In this study, better plant productivity and photosynthetic efficiency in inoculated plants could be possibly because of the growth hormones and other growth-promoting factors produced by the endophytes. Among the 17 endophytic cultures selected for *in vivo* greenhouse trial, two cultures namely *B. subtilis* (A5) and *K. aerogenes* (Y) significantly enhanced the Bacoside A content. Both *B. subtilis* and *K. aerogenes* have been previously reported to have beneficial impact on the plant fitness and health (Berg, 2009; Mukherjee et al., 2020). Keeping in mind the beneficial attributes of the application of synergistic combination of microbes in MAPs (Ray et al., 2019; Tripathi et al., 2020), the study was finally aligned toward evaluation of combinatorial effects of both the cultures with respect to single inoculation. In this investigation, nitrogen fixed by *K. aerogenes* and the synergistic combination resulted in an increase in the concentration of nitrogen in the plant while non-significant increment was observed in uptake of potassium. The increased nitrogen content which is reported to be responsible for influencing the vegetative and the reproductive status of the plants was positively correlated with the herb yield, as well the nitrogen fixation test.

The beneficial microbes induce complex metabolic changes at cellular level which fine-tune the various pathways occurring inside the plant cell for making a “win–win” situation for the plants (Singh et al., 2013; Gupta et al., 2017). The interplay between the reactive oxygen species, different metabolic pathways, and antioxidant status is essential for successful colonization of an endophyte which ultimately decides the fate of the plants (Waszczak et al., 2018). Previously, inoculation with beneficial group of microbes has shown promising leads in regulating the reactive oxygen species levels, thereby helping plants to perform its usual activities both in normal and adverse conditions (Hamilton et al., 2012; Zhou et al., 2016). In our study, significant enhancement was observed in SOD enzyme and total phenolic content which is in concurrence with a study carried out by Huang et al. (2007) who observed high level of production of antioxidants by 292 endophytic morphotypes isolated from 29 plant diverse plant families. One interesting observation was recorded that single inoculation of *B. subtilis* significantly enhanced the total phenolic content which correlated well with the enhanced lignification pattern in the stem of treated plants. The enhanced defensive state of plants treated with *B. subtilis* is in line with the previous observations where microbes have shown encouraging results in terms of enhancing the plant immunity (Van Wees et al., 2008; Yu et al., 2022). Contrary to the above findings, significant upregulation in the bacoside biosynthetic pathway genes was observed in synergistic microbial combination treatment. The results suggested upregulation in phenylpropanoid pathway genes upon inoculation with *B. subtilis*, while the synergistic combination specifically upregulated the genes of bacoside biosynthetic pathway pointing toward the mutualistic effect of both the microbes.

The precursors of all kinds of isoprenoids produced by plants are synthesized by two independent pathways: The MVA pathway occurs in the cytoplasm, while the MEP pathway functions in the plastids (Vranová et al., 2013). The MVA pathway starts with acetyl-coenzyme A through which sequence of events is converted to isopentenyl diphosphate, an intermediate molecule of both MVA and MEP pathways. Our findings revealed upregulation in bacoside biosynthesis pathway genes like *HMGCR* maximally in treatment having synergistic combination of microbes. It is speculated that upregulation in the above gene might have provided pool of substrates for proper functioning of the lower MVA pathway genes like *PMVK*, *FDPS*, *SQS*, and β*- AS* genes which finally yielded higher amount of bacosides as confirmed by HPLC analysis. Our results are in accordance with a recent study carried out by Chen et al. (2021) who showed upregulation in expression level of some important genes like *DXS*, *DXR*, *HMGCR*, and *GGPPS* primarily involved in tanshinone biosynthesis pathway upon inoculation with an endophyte *Mucor circinelloids* DF20 in *Salvia miltiorrhiza.* However, contrary to the above findings, inoculation of *B. subtilis* singly significantly triggered the expression of phenylpropanoid pathway genes. The synthesis of defensive metabolites in the presence of beneficial microbes holds an important strategy adopted by plants to create its niche in its surrounding. A study in support of the above hypothesis reported augmentation in the production of phenylpropanoids and terpenoids in *Atractylodes lancea* upon inoculation with endophytic *Gilmaniella* sp. (Yuan et al., 2016).

Lignin, a constitutively present polymer of phenylpropanoid group in plants, is known to enhance plant cell wall rigidity, thereby promoting mineral transport through the vascular bundles. In addition, it also acts as an important barrier against various biotic stresses. Some reports suggest enhancement in lignification content in response to inoculation of beneficial microbes and their metabolites (Singh et al., 2013; Singh et al., 2016; Patel et al., 2017). Upregulation in transcripts of phenylpropanoid pathway genes like *CCR, CAD, HST*, and *CHS* genes in *B. subtilis* treatment is also in line with the above studies. We hypothesize that the upregulation in the above genes could be the main reason for enhanced lignifications in the above treatment as CAD is the final enzyme responsible for converting sinapaldehyde to sinapyl alcohol which finally leads to formation of syringyl lignin. In addition to the role of primary genes involved in the biosynthetic pathways, the expression of plant secondary metabolites is under the governance of various kinds of transcription factors belonging to diverse families of *bHLH, MYB*, and *WRKY* (Yang et al., 2012; Patra et al., 2013; Gandhi et al., 2015). Enhanced expression of *WRKY 1* in combination treatment is in accordance with previous studies which state the role of *WRKY* transcription factors in regulation of plant development and production of secondary metabolites (Xu et al., 2004; Ma et al., 2009; Suttipanta et al., 2011). Likewise, higher expression of *MYB 1* in *B. subtilis* alone points out toward its role in lignin biosynthesis as reported in previous studies (Ambawat et al., 2013; Miyamoto et al., 2020).

**FIGURE 10:**
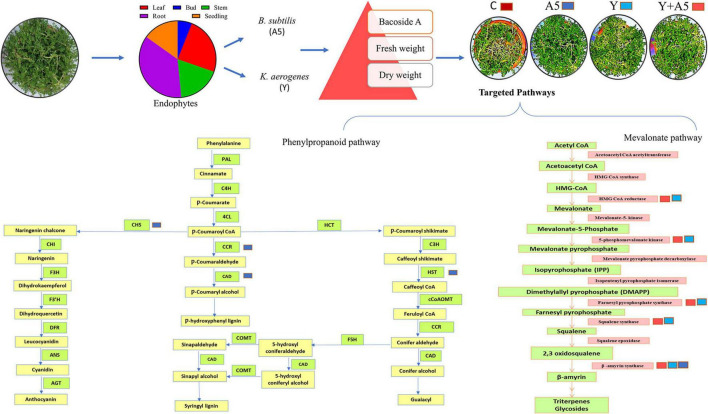
Schematic representation of the impact of endophytic cultures *B. subtilis* (A5) and K. aerogenes (Y) singly as well as in combination on plant fitness and secondary metabolite biosynthetic pathways.

Overall, the results of this study strongly suggest that the deployment of combined inoculation of *B. subtilis* with *K. aerogenes* enhances the transcription of bacoside biosynthetic pathway genes while single inoculation of *B. subtilis* specifically impacted the phenylpropanoid pathway (Figure 10). The leads obtained can be exploited to use specific microbes as per requirement, that is, *B. subtilis* inoculation alone in future can be used for managing biotic/abiotic stresses while combined inoculation of the endophytes could be suggested as a sustainable value added strategy for cultivation of *B. monnieri*. On the whole by using native endophytic microbes, *in planta* production of pharmaceutically important bacosides in *B. monnieri* can be enhanced which can provide non-chemical and cheap alternative for sustainable agriculture for future.

## Data availability statement

The datasets presented in this study can be found in online repositories. The names of the repository/repositories and accession number(s) can be found in the article/Supplementary material.

## Author contributions

AS conceived and designed the experiments and wrote the manuscript. NS, DS, AT, PK, RG, SS, and KS performed the experiments. AS and RKG analyzed the data. All authors read and approved the manuscript.
