# Supplementary material for: Synergism of endophytic Bacillus subtilis and Klebsiella aerogenes modulates plant growth and bacoside biosynthesis in Bacopa monnieri
Source: Front Plant Sci. 2022 Aug 4;13:896856. doi: 10.3389/fpls.2022.896856 (PMC9386127; doi:10.3389/fpls.2022.896856)

**Synergism of endophytic *Bacillus subtilis* and *Klebsiella aerogenes* modulate plant growth and bacoside biosynthesis in *Bacopa monnieri***

Supplementary Table T1 List of primers used in RT-PCR study

| **Sl No** | **Target transcripts** |  | **Primer sequences (5′ to 3′)** |
| --- | --- | --- | --- |
|  | cinnamyl-alcohol dehydrogenase (CAD) |  | Quantitative RT-PCR |
|  |  | F | TCGACAAGCCCGGATTGA |
|  |  | R | CGAAAGCCTTAGCGAATTTCAC |
|  | hydroxymethylglutaryl-CoA reductase  (HMGR) | F | CTTTCCCCTGCCTTCAATCC |
|  |  | R | ATGGACGTCATCGGAATCTCA |
| 1. 14 | beta-amyrin synthase (β-AS) | F | CCAAGCATAGCCCATGATGTC |
|  |  | R | CGAATCCTGTCCAAGCATGA |
| 1. 18 | shikimate O-hydroxycinnamoyltransferase (HST) | F | GGAGCTCCGCAGCCTAATTC |
|  |  | R | TGCGTTACCTGCAACACCAA |
| 1. 22 | chalcone synthase (CHS) | F | TCAACCTCCGCCAGGAAAT |
|  |  | R | CCCATTCTTCAATCGCCTTCT |
| 1. 23 | squalene synthase (SQS) | F | ATGACCTGAGGAATTGCACAAA |
|  |  | R | GCATGTCGAAGATTGCATCAA |
| 1. 24 | farnesyl diphosphate synthase (FDPS) | F | TTCCTGTTATTGAAGAATCTCCAAAA |
|  |  | R | CAAGTACAATTGAGACAAATAGCATATGA |
| 1. 28 | 5-phosphomevalonate kinase (PMVK) | F | AGGTGGCTTTGACGCAGTCT |
|  |  | R | ACGTTGAGCGAACTCCACACT |
| 1. 30 | Cinnamoyl-CoA reductase (CCR) | F | TGGTTTGAGAATTTGTACGGTTTTT |
|  |  | R | GGAGCATTAACTTTACCTTTCATGTG |
|  | MYB-1 | F | TCCAAAAGATTACTATCCCAAGAAGAATA |
|  |  | R | TAGCCTTTCTGCCCCACAAA |
|  | MYB-2 | F | TGGAGATCAAACCCCTGGAA |
|  |  | R | CAAGCATTAGCCAAGTCGATGA |
|  | BHLH-1 | F | TTATTCTATCTCCTAACTTCTCCTTTCTCA |
|  |  | R | GGGTATCTAAGAAGGCTAGGGTTCA |
|  | WRKY-1 | F | GGAAAAAAGGCACCAGAACAAG |
|  |  | R | GAATGTGTCACGGGATGTGTTG |
|  |  | R | CCGAAAATATTCAACACCATCCTA |
|  | MYB-3 | F | TGTTATTCTTGGATCTTGATGGCTTA |
|  |  | R | GCCGCAATAGCATCAGTTACAG |

Supplementary Table T2 List of endophytes isolated from *Bacopa monnieri*

| S. No. | Plant Part | Isolate | Bacteria/Fungi |
| --- | --- | --- | --- |
| 1 | Bud | G1 | Bacteria |
|  |  | G2 | Bacteria |
| 2 | Leaf | A | Bacteria |
|  |  | C | Bacteria |
|  |  | D | Bacteria |
|  |  | J | Fungi |
|  |  | L | Bacteria |
|  |  | R | Bacteria |
|  |  | S | Bacteria |
|  |  | A5 | Bacteria |
|  |  | A8 | Bacteria |
|  |  | A10 | Fungi |
|  |  | F2 | Fungi |
|  |  | F3 | Fungi |
|  |  | F4 | Fungi |
| 3 | Stem | B | Bacteria |
|  |  | E | Bacteria |
|  |  | F | Bacteria |
|  |  | H | Fungi |
|  |  | K | Fungi |
|  |  | Q | Bacteria |
|  |  | V | Fungi |
|  |  | W | Fungi |
|  |  | X | Fungi |
|  |  | A3 | Bacteria |
|  |  | A7 | Bacteria |
|  |  | A12 | Fungi |
|  |  | A13 | Fungi |
|  |  | A15 | Fungi |
|  |  | F1 | Fungi |
| 4 | Root | I | Bacteria |
|  |  | M | Bacteria |
|  |  | N | Bacteria |
|  |  | O | Bacteria |
|  |  | P | Bacteria |
|  |  | T | Bacteria |
|  |  | U | Bacteria |
|  |  | Y | Bacteria |
|  |  | Z | Bacteria |
|  |  | A1 | Bacteria |
|  |  | A9 | Bacteria |
|  |  | A11 | Bacteria |
| 5 | Seedlings | A2 | Bacteria |
|  |  | A4 | Bacteria |
|  |  | A6 | Bacteria |
|  |  | A14 | Bacteria |
|  |  | A16 | Bacteria |

Supplementary Table T3 Colony morphology of the isolated bacterial endophytes

| S. No. | | Isolate | Form | Margin | Elevation | Density | Colour | Texture |
| --- | --- | --- | --- | --- | --- | --- | --- | --- |
| 1 | A | | Irregular | Curled | Umbonate | Opaque | Off-white | Dull, rough |
| 2 | B | | Irregular | Entire | Crateriform | Opaque | Off-white | Smooth, slimy, glistening |
| 3 | C | | Irregular | Undulate | Raised | Opaque | Greenish-yellow | Dry, rough |
| 4 | D | | Irregular | Lobate | Raised | Opaque | Off-white | Smooth, slimy |
| 5 | E | | Irregular | Lobate | Raised | Opaque | Off-white | Dry |
| 6 | F | | Spindle | Undulate | Flat | Opaque | Off-white | Dry |
| 7 | G1 | | Spindle | Entire | Raised | Opaque | Yellow | Smooth, slimy, glistening |
| 8 | G2 | | Irregular | Lobate | Raised | Opaque | Off-white | Dry, rough |
| 9 | I | | Irregular | Undulate | Umbonate | Opaque | White | Dry, rough |
| 10 | L | | Punctiform | Entire | Raised | Opaque | Neon yellow | Smooth, dry |
| 11 | M | | Circular | Undulate | Umbonate | Opaque | Off white | Rough |
| 12 | N | | Punctiform | Entire | Raised | Watery opaque | White | Slimy, smooth |
| 13 | O | | Irregular | Undulate | Flat | Opaque | Off-white | Dry, rough |
| 14 | P | | Spindle | Undulate | Raised | Opaque | White | Dry, smooth |
| 15 | Q | | Circular | Entire | Convex | Opaque | Cream | Smooth, slimy, glistening |
| 16 | R | | Circular | Entire | Convex | Opaque | Off-white | Rough |
| 17 | S | | Irregular | Undulate | Raised | Opaque | Off-white | Slimy |
| 18 | T | | Irregular | Undulate | Raised | Opaque | Off-white | Slimy |
| 19 | U | | Irregular | Lobate | Flat | Opaque | Off-white | Slimy, smooth |
| 20 | Y | | Irregular | Undulate | Flat | Opaque | Off-white | Dry, rough |
| 21 | Z | | Irregular | Lobate | Raised | Opaque | Off-white | Dry, smooth |
| 22 | A1 | | Irregular | Entire | Convex | Opaque | Off-white | Glistening, slimy |
| 23 | A2 | | Circular | Undulate | Convex | Opaque | Cream | Dull, slimy |
| 24 | A3 | | Irregular | Undulate | Flat | Opaque | Off-white | Dry, rough |
| 25 | A4 | | Circular | Undulate | Raised | Opaque | Cream | Dry, rough |
| 26 | A5 | | Irregular | Entire | Raised | Opaque | Off-white | Glistening, rough |
| 27 | A6 | | Irregular | Undulate | Raised | Opaque | Cream | Dry, rough |
| 28 | A7 | | Irregular | Undulate | Raised | Opaque | Cream | Smooth, dull |
| 29 | A8 | | Circular | Entire | Umbonate | Opaque | Cream | Smooth, dull |
| 30 | A9 | | Irregular | Undulate | Flat | Opaque | Off-white | Dry, rough |
| 31 | A11 | | Irregular | Undulate | Raised | Opaque | Off-white | Dull, slimy |
| 32 | A14 | | Irregular | Undulate | Convex | Opaque | Off-white | Dull, smooth |
| 33 | A15 | | Irregular | Undulate | Convex | Opaque | Off-white | Dull, smooth |
| 34 | A16 | | Spindle | Undulate | Convex | Opaque | Off-white | Dull, smooth |

Supplementary Table T4 Colony morphology of the isolated fungal endophytes

| S.No. | Isolate | Colour  Front Back | | Texture |
| --- | --- | --- | --- | --- |
| 1 | H | Slate-green | Brownish white | Rough, powdery |
| 2 | J | Grey | Brown | Cottony |
| 3 | K | Black | Cream | Dry, rough |
| 4 | V | Dark-green | Black | Dry, sporulating |
| 5 | W | Black | Black | Dry |
| 6 | X | Black | Black with white margin | Cottony with black spores |
| 7 | A10 | Dark-green | Black | Velvety |
| 8 | A12 | Dark-green | Brown | Wrinkled, velvety |
| 9 | A13 | Brown | Brown | Velvety |
| 10 | F1 | Dark-green | Black | Velvety |
| 11 | F2 | White with green centre | White | Velvety |
| 12 | F3 | Dark-green | Black | Velvety |
| 13 | F4 | Black with white margin | Black | Velvety |

Supplementary Table T5 Phosphate solubilization index

| S. No. | Isolate | Phosphate Solubilization Zone (diameter in mm) | Colony diameter (mm) | Phosphate Solubilization Index (SI) |
| --- | --- | --- | --- | --- |
| 1 | G1 | + (11 mm) | 5 mm | 1.2 |
| 2 | H | + (25 mm) | 17 mm | 0.5 |
| 3 | L | + (8 mm) | 5 mm | 0.6 |
| 4 | Q | + (13 mm) | 7 mm | 0.86 |

Supplementary Table T6 Zinc solubilization index and efficiency

| S.No. | Isolate | Zinc Solubilization Zone (diameter in mm) | Colony diameter (mm) | Zinc solubilization Index (ZSI) | Zinc Solubilization Efficiency (ZSE) |
| --- | --- | --- | --- | --- | --- |
| 1 | G1 | 8 mm | 3 mm | 1.67 | 266.67 |

Supplementary Figure S1 Effect of selected endophytes on plant growth parameters


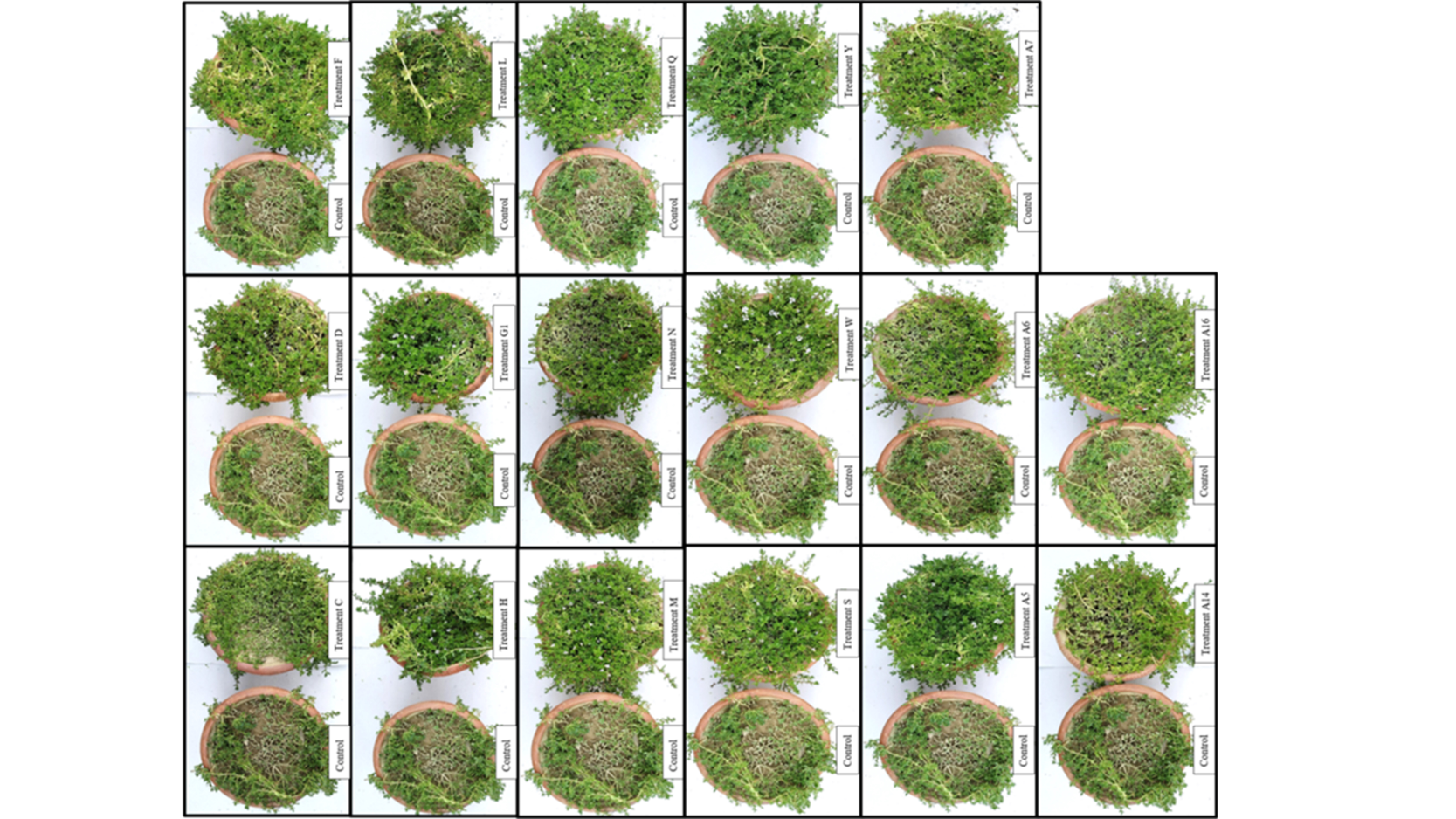

Supplement: Supplementary file 1 [file Data_Sheet_1.docx]
